# Extra virgin olive oil use is associated with improved post-prandial blood glucose and LDL cholesterol in healthy subjects

**DOI:** 10.1038/nutd.2015.23

**Published:** 2015-07-20

**Authors:** F Violi, L Loffredo, P Pignatelli, F Angelico, S Bartimoccia, C Nocella, R Cangemi, A Petruccioli, R Monticolo, D Pastori, R Carnevale

**Affiliations:** 1 Department of Internal Medicine and Medical Specialties, Sapienza University of Rome, Rome, Italy; 2 Department of Public Health and Infectious Diseases, Sapienza University of Rome, Rome, Italy; 3 AFC Patrimonio Servizi e furniture UO ristorazioni, Policlinico Umberto I, Rome, Italy; 4 Department of Medico-Surgical Sciences and Biotechnologies, Sapienza University of Rome, Latina, Italy

## Abstract

**Objectives:**

Extra virgin olive oil (EVOO) is a key component of the Mediterranean diet and seems to account for the protective effect against cardiovascular disease. However, the underlying mechanism is still elusive.

**Design:**

We tested the effect of EVOO, added to Mediterranean-type meal, on post-prandial glycemic and lipid profile.

**Subjects:**

Post-prandial glycemic and lipid profile were investigated in 25 healthy subjects who were randomly allocated in a cross-over design to a Mediterranean-type meal added with or without 10 g EVOO (first study), or Mediterranean-type meal with EVOO (10 g) or corn oil (10 g; second study). Glycemic profile, which included glucose, insulin, dipeptidyl-peptidase-4 (DPP-4) protein and activity, glucagon-like peptide-1 (GLP-1) and glucose-dependent insulinotropic polypeptide (GIP), and lipid profile, which included, low-density lipoprotein (LDL) cholesterol (LDL-C), oxidized LDL (ox-LDL), triglycerides and high-density lipoprotein (HDL) cholesterol (HDL-C), were analyzed before and 2 h after the meal.

**Results:**

In the first study, 2 h after meal, subjects who assumed a meal with EVOO had significantly lower blood glucose (*P*<0.001), DPP-4 protein (*P*<0.001) and activity (*P*<0.001), LDL-C (*P*<0.001) and ox-LDL (*P*<0.001) and higher insulin (*P*<0.05), GLP-1 (*P*<0.001) and GIP (*P*<0.05) compared with those without EVOO. The second study showed that compared with corn oil, EVOO improved both glycemic and lipid profile. Thus, a significantly smaller increase of glucose (*P*<0.05), DPP4 protein (*P*<0.001) and activity (*P*<0.05) and higher increase of insulin (*P*<0.001) and GLP-1 (*P*<0.001) were observed. Furthermore, compared with corn oil, EVOO showed a significantly less increase of LDL-C (*P*<0.05) and ox-LDL (*P*<0.001).

**Conclusions:**

We report for the first time that EVOO improves post-prandial glucose and LDL-C, an effect that may account for the antiatherosclerotic effect of the Mediterranean diet.

## Introduction

Mediterranean diet is the golden standard for healthy nutrition and is associated with reduced risk of cardiovascular events.^1^ Traditionally, Mediterranean diet is characterized by high intake of fruits, vegetables, cereals, fish and moderate wine consumption, with scarce intake of dairy products and red meat. Most important, the health benefits of Mediterranean diet have been attributed to the high intake of monounsaturated fat, mostly represented by extra virgin olive oil (EVOO). Thus, recent results from the PREDIMED study showed that EVOO added to the Mediterranean diet reduces the risk of cardiovascular events compared with controls.^1^ Among the mechanism(s) potentially attributable to the antiatherosclerotic effect, the prevention of new-onset diabetes might have a role, but the biologic plausibility of such positive association is still unclear. Prevention of diabetes might be attributable to the antioxidant property of EVOO;^2^ thus, oxidative stress seems to be implicated in β-cells dysfunction and eventually diabetes.^3^ Furthermore, oxidative stress is responsible for activation of dipeptidyl-peptidase-4 (DPP-4),^4^ which cleaves incretins downregulating insulin secretion.^5^ We have recently demonstrated that a Mediterranean-type meal supplemented with EVOO is associated with reduced post-prandial oxidative stress generated by NOX2, the catalytic subunit of NADPH oxidase.^6^ On the basis of this finding, we speculated that EVOO, added to Mediterranean-type meal, could improve post-prandial glycaemic control *via* an oxidative stress-mediated mechanism. In addition, we investigated if EVOO had any effect on lipid profile including analysis of low-density lipoprotein (LDL)-cholesterol (LDL-C), triglycerides and high-density lipoprotein (HDL)-cholesterol (HDL-C).

## Subjects and methods

Twenty-five healthy subjects (HS; 12 males and 13 females) gave informed consent to participate in the interventional study, which was performed between January 2013 and March 2013. Study methodology, clinical and demographics characteristics of HS and Mediterranean-type lunch have been previously reported.^6^ Briefly, a first study consisted in randomizing 25 HS were to receive a typical Mediterranean lunch including or not 10 g of EVOO (Lago dei Papi, Viterbo, Italy; see Table 1, Supplementary Data) in a cross-over design; there was an interval of 30 days between the two phases of the study. Mediterranean-type lunch consisted of pasta (100 g), chicken breast (150 g), salad (80 g), bread (80 g), apple (200 g) for a total of 894 calories. After 1 month from the end of the first study the same subjects (*n*=25) participated to a second study in which they were randomly allocated to receive a lunch with EVOO (10 g) or corn oil (10 g) in a cross-over design. There was an interval of 30 days between the two phases of the study. For each phase of the study, a blood sample was taken before (at 1300 hours) and 2 h after the lunch. Every blood determination was performed blind. None of the participants were receiving antioxidants supplements or statin. The study was conformed to the ethical guidelines of the 1975 Declaration of Helsinki and was approved by the Ethical Committee of Sapienza University.

### Laboratory analysis

Blood concentration of glucose, insulin, total GLP1 (7–36 and 9–36 peptides), total GIP, DPP-4 protein and activity, ox-LDL, LDL-C, triglycerides and HDL-C were measured by ELISA Kit (Sigma Aldrich, DRG International, Cusabio, Boster). Oil analysis were described in Supplementary Data.

### Sample size

As regards the interventional cross-over study, we computed the minimum sample size with respect to a two-tailed one-sample Student's *t*-test, considering as (i) glucose variation to be detected between extra virgin oil and corn oil treatment |*δ*|≥15 mg dl^−1^, (ii) s.d. of the paired differences s.d.=15 mg dl^−1^, (iii) type I error probability *α*=0.05 and power 1−*β*=0.90. This resulted in a minimum sample size of 10 per group.

### Statistical methods

Categorical variables are reported as counts (percentage) and continuous variables as means±s.d. unless otherwise indicated. Independence of categorical variables was tested by *χ*^2^-test. Comparisons between groups were carried out by Student's *t*-test and were replicated as appropriate with nonparametric tests (Kolmogorov–Smirnov (*z*) test in case of nonhomogeneous variances as verified by Levene's test).

The cross-over study data were analyzed for the assessment of treatment and period effects, by performing a split-plot ANOVA with one between-subject factor (treatment sequence) and two within-subject factors (period 1 vs 2; pre- vs post-treatment). The analysis was performed separately to compare a meal with and without EVOO and a meal with EVOO vs corn oil. The full model was considered, allowing for the assessment of all main effects and interactions. Pairwise comparisons were corrected by the Bonferroni test; results were expressed as means±s.e. Bivariate analysis was performed by Spearman rank correlation test. A value of *P*<0.05 was considered statistically significant. All analyses were carried out with SPSS V.18.0 (SPSS Statistics v. 18.0, SPSS Inc., Chicago, IL, USA).

## Results

### EVOO vs non-EVOO effect on post-prandial glycaemic control

**Figure 1:**
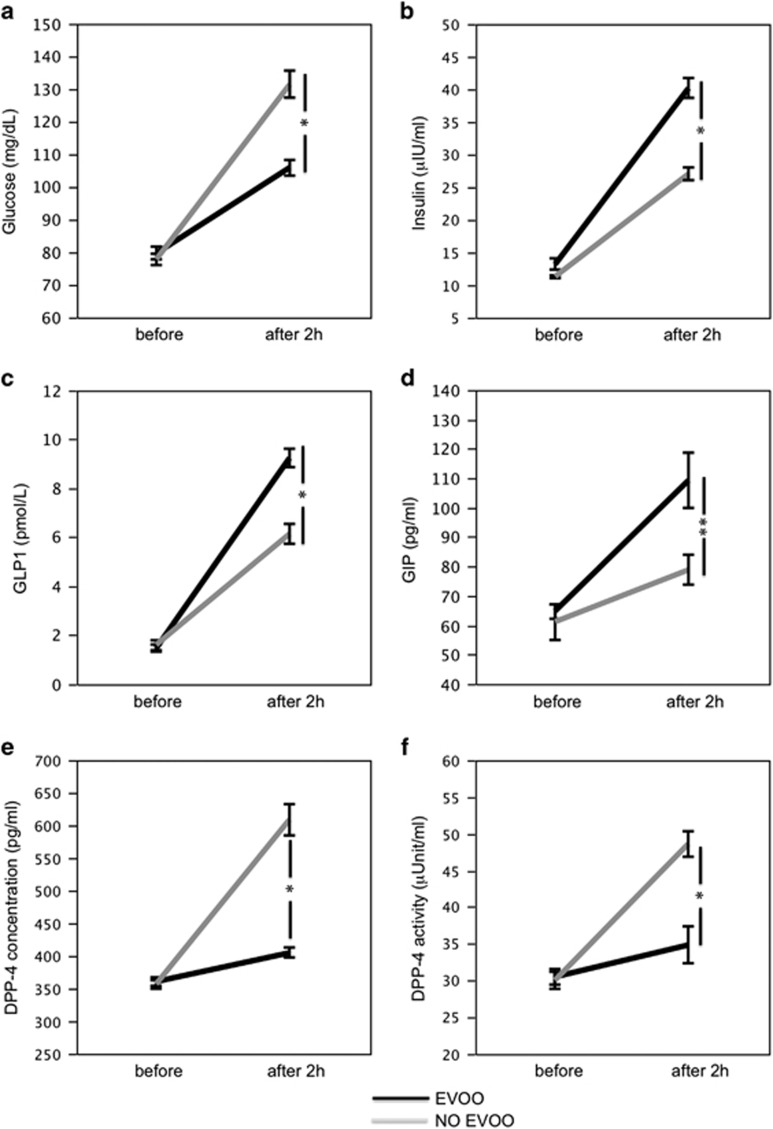
Interventional study: blood glucose (**a**), insulin (**b**), GLP1 (**c**), GIP (**d**), DPP-4 concentration (**e**) and DPP-4 activity (**f**) before and after 2 h of a meal with (black line) or without (gray line) extra virgin olive oil (EVOO), *P<0.001, **P<0.05.

In the first study, we compared the effect of EVOO added or not to a Mediterranean-type lunch. At baseline, no differences in blood variables were detected (Figure 1). Two hours after a Mediterranean-type lunch, a significant difference for treatments (meal with vs without EVOO) was found with respect to glucose (F=27.8, *P*<0.001; Figure 1a), insulin (F=38.3, *P*<0.001; Figure 1b), GLP1 (F=31.0, *P*<0.001; Figure 1c), GIP (F=5.3, *P*=0.025; Figure 1d), DPP-4 concentration (F=62.9, *P*<0.001; Figure 1e) and DPP-4 activity (F=16.0, *P*<0.001; Figure 1f).

In particular, compared with baseline, when a meal not containing EVOO was given, glucose concentration and insulin increased significantly (Figures 1a and b; Table 1). Conversely, compared with baseline, in subjects given a meal containing EVOO, a less increase of blood glucose and a more marked increase of blood insulin were detected (Figures 1a and b; Table 1).

Analysis of incretins' secretion showed significant differences between the meal with and without EVOO. Thus, incretins increased more significantly in subjects supplemented with EVOO compared with those who did not receive it (Figures 1c and d; Table 1); furthermore, DPP-4 protein and activity showed a lower increase after meal with EVOO (Figures 1e and f; Table 1).

### EVOO vs non-EVOO effect on post-prandial lipid profile

**Figure 2:**
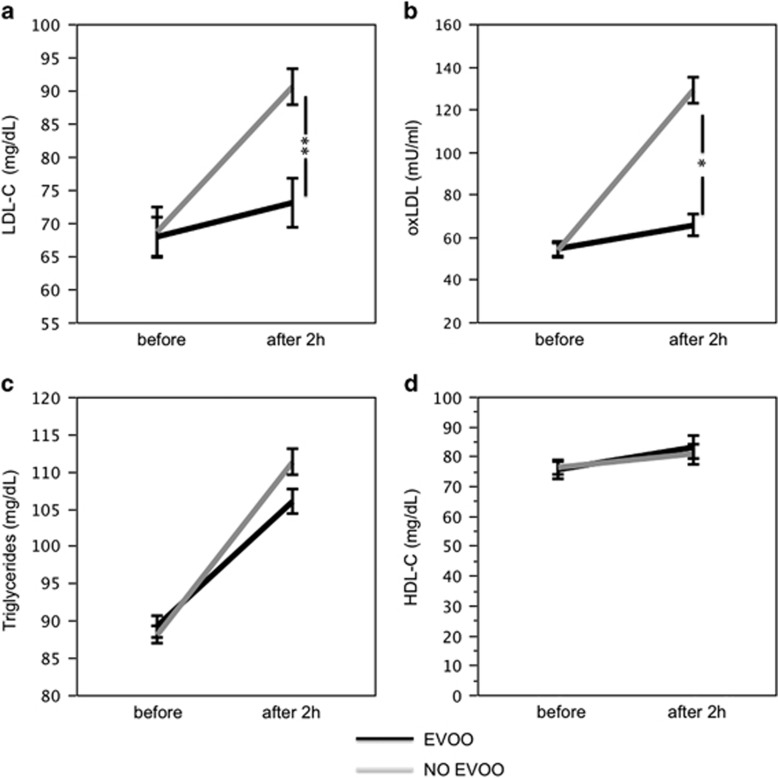
Interventional study: LDL-C (**a**), ox-LDL concentration (**b**), triglycerides (**c**) and HDL-C (**d**) before and after 2 h of a meal with (black line) or without (gray line) extra virgin olive oil (EVOO), *P<0.001, **P<0.05.

At baseline, no differences of blood variables were detected (Figure 2). Two hours after a Mediterranean-type lunch, a significant difference for treatments (meal with vs without EVOO) was found with respect to LDL-C (F=6.4, *P*=0.014; Figure 2a) and ox-LDL (F=144.0, *P*<0.001; Figure 2b). No significant difference was found with respect to triglycerides and HDL-C (Figures 2c and d). In particular, compared with baseline, when a meal not containing EVOO was given, LDL-C, ox-LDL and triglycerides increased significantly, whereas HDL-C did not change (Figures 2a–d; Table 1). Conversely, compared with baseline, in subjects given a meal containing EVOO, a significantly less increase of LDL-C and ox-LDL was detected, whereas triglycerides and HDL-C did not change (Figures 2a–d; Table 1). Δ of ox-LDL correlated with **Δ** of glycaemia (*R*_S_: 0.501; *P*<0.001), Δ of insulin (*R*_S_:-0.492; *P*<0.001), Δ of DPP-4 activity (*R*_S_: 0.467; *P*=0.001), Δ of DPP-4 concentration (*R*_S_=0.508; *P*<0.001) and Δ of GLP1 (*R*_S_: −0.353; *P*=0.012). Δ of DPP-4 activity correlated with serum glucose (*R*_S_: 0.503; *P*=0.001).

### EVOO and corn oil effect on post-prandial glycaemic and lipid profile

**Figure 3:**
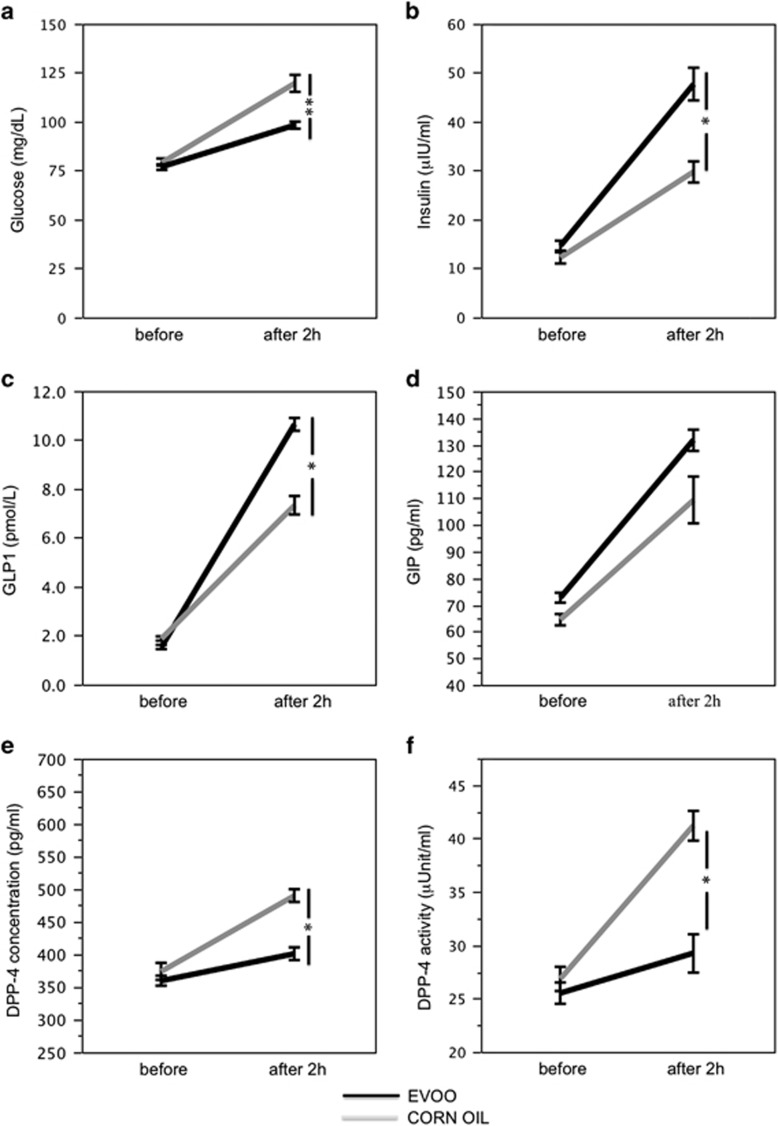
Interventional study: blood glucose (**a**), insulin (**b**), GLP1 (**c**), GIP (**d**), DPP-4 concentration (**e**) and DPP-4 activity (**f**) before and after 2 h of a meal with extra virgin olive oil (EVOO; black line) or corn oil (gray line), *P<0.001, **P<0.05.

**Figure 4:**
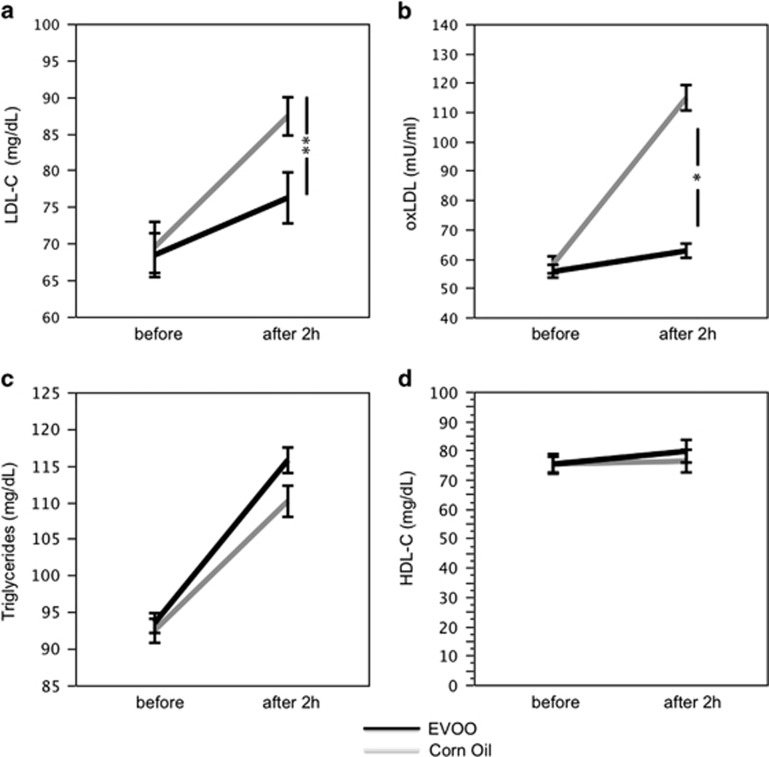
Interventional study: LDL-C (**a**), ox-LDL concentration (**b**), Triglycerides (**c**) and HDL-C (**d**) before and after 2 h of a meal with extra virgin olive oil (EVOO; black line) or corn oil (gray line), *P<0.001, **P<0.05.

When we compared the effect of a meal containing EVOO or corn oil, a significant difference for treatments with respect to glucose (F=13.9, *P*=0.01; Figure 3a), insulin (F=14.4, *P*<0.001; Figure 3b), GLP1 (F=55.6, *P*<0.001; Figure 3c), DPP-4 concentration (F=23.0, *P*<0.001; Figure 3e), DPP-4 activity (F=12.5, *P*=0.001; Figure 3f), LDL-C (F=4.1, *P*<0.05; Figure 4a) and ox-LDL (F=67.5, *P*<0.001; Figure 4b) was detected. No significant difference for treatments was found with respect to GIP, triglycerides and HDL-C (Figures 3d and 4c and d).

In particular, after 2 h from lunch, in subjects given a meal containing corn oil, glucose concentration, insulin, GLP1, GIP, DPP-4 concentration and DPP-4 activity increased significantly (Figure 3a–f; Table 2). These changes were associated with a significant increase of LDL-C, ox-LDL and triglycerides, whereas HDL-C did not significantly change (Figure 4a–d; Table 2).

Compared with corn oil, subjects given a meal containing EVOO showed a significantly less increase of blood glucose, DPP-4 concentration and activity and a more marked increase of blood insulin, GLP1, GIP (Figure 3a–f; Table 2). Concerning lipid profile, subjects given EVOO showed a significantly smaller increase of LDL-C and ox-LDL, whereas triglycerides showed a non-significant trend toward reduction (Figures 4a–d; Table 2).

## Discussion

This is the first study demonstrating that a Mediterranean-type meal supplemented with EVOO has a beneficial effect on post-prandial glycemic and lipid profile by decreasing blood glucose, LDL-C and ox-LDL.

There is a growing body of evidence to suggest that post-prandial changes of glycemic and lipid profile might have deleterious effect on vascular disease by promoting and/or aggravating the atherosclerotic process.^7, 8^ Moreover, post-prandial glucose has been associated with an higher incidence of cardiovascular events in patients with^9^ and without diabetes.^10^ Thus, blunting or minimizing post-prandial glycemic and lipid profile could have a positive impact in the progression of atherosclerosis.^11^ Observational and interventional studies consistently demonstrated a potentially beneficial effect of EVOO on the atherosclerotic process,^1, 12, 13^ but the underlying mechanism is still undefined. Data regarding the impact of EVOO on post-prandial glycemic and lipid profile are still lacking. The novelty of the present study is in the demonstration that EVOO improves the post-prandial glycemic control by lowering and increasing blood glucose and insulin, respectively. Post-prandial glycemic control is regulated by incretins, which upregulate insulin secretion and in turn lowers blood glucose. Incretins such as GLP1 and GIP are secreted by distal small intestine in response to its stimulation, bind receptors in the endocrine pancreas so eliciting insulin secretion and lowering post-prandial blood glucose and are rapidly inactivated by DPP-4.^14, 15^ Our approach was to evaluate whether EVOO may stimulate intestinal cells and trigger endogenous incretin release. Analysis of GLP-1 and GIP after Mediterranean-type lunch demonstrated that supplementation with EVOO was associated with an increase of both incretins coincidentally with a decrease of DPP-4 activity suggesting that EVOO behaves as a DPP-4 inhibitor. The antioxidant effect of EVOO^6^ may account for the improved post-prandial glycaemia as oxidative stress is implicated in incretin secretion and metabolism. Oxidative stress affects, in fact, insulin signaling cascade, leading to insulin resistance^3^ and cumulative hyperglycemia and resultant AGE-induced ROS generation^16^ might impair the incretins' effects via elevation of circulating DPP-4 concentration.^4^ Of note, we found that, compared with control, post-prandial ox-LDL increase was almost blunted by EVOO and paralleled DPP-4 activity changes with a direct correlation between these two variables, reinforcing the concept that oxidative stress upregulates DPP-4 activity^4^ and eventually inhibits insulin secretion. Among the EVOO components, oleuropein would be likely to downregulate NOX-2-derived oxidative stress,^6^ but further study is necessary to investigate if the polyphenol component of EVOO also have a role.

An unexpected finding of the study was the improvement of lipid profile observed after EVOO administration. Thus, post-prandial serum LDL-C was significantly reduced compared with control suggesting a lipid-lowering effect of EVOO. This effect seems to be specific for LDL-C, as neither triglycerides nor HDL-C were modified compared with control. The positive impact of EVOO on post-prandial LDL-C is more difficult to interpret. We have not data that may help to explain if such beneficial effect depends on a specific EVOO interference with cholesterol absorption in the gastrointestinal tract or on a direct activity of EVOO on cholesterol biosynthesis. This issue deserves further investigation.

To investigate if these changes on serum glucose and LDL-C were peculiar for EVOO, we compared EVOO versus corn oil with regard to the changes of post-prandial glycemic and lipid profile. Also this experiment confirmed the beneficial effect of EVOO as it significantly improved both glycemic and lipid profile compared with corn oil so suggesting that its components may favorably influence glucose and cholesterol metabolism.

The study has limitation and implication. We have not data specifically addressing insulin secretion because early serum glucose and insulin increment after lunch are not available.^17^ The exact mechanism through which EVOO downregulates DPP-4 has not been addressed in the present study and should be investigated. It is possible that EVOO downregulates NOX2-derived oxidative stress,^6^ but it cannot be excluded that post-prandial LDL-C lowering may also have a role.^18^ We cannot exclude that corn oil may have positive effect when compared with control as this issue was not investigated in the present study. The study has been performed in healthy subjects and, therefore, further study is necessary to see whether EVOO has such beneficial effect in patients with diabetes or dyslipidemia. Finally it remains to establish whether the changes observed with EVOO persist chronically.

While our findings provide a potential explanation for the inverse association between Mediterranean diet and diabetes risk, as GLP-1 may reduce cellular apoptosis in the pancreatic β-cells and promote β-cell proliferation,^15^ the positive effect of EVOO on insulin and LDL-C opens new avenues to counteract the potentially deleterious effects on vascular function related to post-prandial spikes in glucose and lipid.^19^

In conclusion, EVOO has beneficial effect on post-prandial glycemic and LDL-C profile so providing a novel insight into the mechanism potentially accounting for the antiatherosclerotic effect of the Mediterranean diet.

## Figures and Tables

**Table 1:** Post-prandial effect of a meal with and without EVOO on selected variables

*Variables*	*Before*	*After 2 h*	P*-value*
*Meal with EVOO*			
Glucose (mg dl^−1^)	79.7±9.7	105.9±12.1	<0.001
Insulin (μIU ml^−1^)	13.2±4.2	40.8±7.8	<0.001
GIP (pg ml^−1^)	64.8±12.0	109.3±48.0	<0.001
GLP1 (pmol l^−1^)	1.4±0.7	9.2±1.7	<0.001
DPP-4 concentration (pg ml^−1^)	360.8±36.4	404.9±39.9	<0.05
DPP-4 activity (μU ml^−1^)	30.5±4.9	34.8±12.2	<0.05
HDL-C (mg dl^−1^)	75.5±14.3	83.0±18.8	NS
Triglycerides (mg dl^−1^)	89.2±7.1	105.9±8.2	<0.001
LDL-C (mg dl^−1^)	67.9±15.0	73.0±18.0	<0.05
Ox-LDL (mU ml^−1^)	54.4±18.3	65.4±24.7	NS
			
*Meal without EVOO*			
Glucose (mg dl^−1^)	77.8±8.3	131.4±20.9	<0.001
Insulin (μIU ml^−1^)	11.3±1.4	27.0±5.0	<0.001
GIP (pg ml^−1^)	61.2±30.0	78.8±24.6	<0.001
GLP1 (pmol l^−1^)	1.6±0.9	6.1±1.8	<0.001
DPP-4 concentration (pg ml^−1^)	357.0±33.0	608.9±115.0	<0.001
DPP-4 activity (μU ml^−1^)	30.0±5.9	48.6±8.5	<0.001
HDL-C (mg dl^−1^)	76.2±12.2	80.8±17.2	NS
Triglycerides (mg dl^−1^)	88.0±5.7	111.0±8.6	<0.001
LDL-C (mg dl^−1^)	68.6±19.2	90.5±13.1	<0.001
Ox-LDL (mU ml^−1^)	53.7±17.0	128.9±29.2	<0.001

Abbreviations: DPP-4, Dipeptidyl peptidase-4; EVOO, extra virgin olive oil; GIP, glucose-dependent insulinotropic polypeptide; GLP1, glucagon-like peptide-1; LDL, low-density lipoprotein; NS, not significant; Ox-LDL, oxidized LDL; LDL-C, LDL-cholesterol; HDL-C, HDL-cholesterol.

**Table 2:** Post-prandial effect of a meal with EVOO or corn oil on selected variables

*Variables*	*Before*	*After 2 h*	P*-value*
*Meal with EVOO*			
Glucose (mg dl^−1^)	77.0±7.8	98.2±9.1	<0.001
Insulin (μIU ml^−1^)	14.6±5.4	47.6±16.6	<0.001
GIP (pg ml^−1^)	72.6±8.9	131.8±19.9	<0.001
GLP1 (pmol l^−1^)	1.5±0.4	10.6±1.2	<0.001
DPP-4 concentration (pg ml^−1^)	359.6±33.8	401.0±49.8	<0.001
DPP-4 activity (μU ml^−1^)	25.5±4.9	29.2±8.9	<0.05
HDL-C(mg dl^−1^)	75.3±16.1	79.6±19.8	NS
Triglycerides (mg dl^−1^)	92.5±8.1	110.1±10.6	<0.001
LDL-C (mg dl^−1^)	68.4±14.8	76.2±17.1	<0.001
Ox-LDL (mU ml^−1^)	55.6±10.9	62.6±11.2	NS
			
*Meal with corn oil*			
Glucose (mg dl^−1^)	79.0±8.5	119.7±21.1	<0.001
Insulin (μIU ml^−1^)	12.2±5.7	29.7±10.7	<0.001
GIP (pg ml^−1^)	64.4±10.0	109.2±44.4	<0.001
GLP1 (pmol l^−1^)	1.8±0.5	7.6±1.7	<0.001
DPP-4 concentration (pg ml^−1^)	373.8±67.1	490.0±49.3	<0.001
DPP-4 activity (μU ml^−1^)	26.8±5.9	41.2±6.9	<0.001
HDL-C (mg dl^−1^)	75.3±13.1	76.3±19.1	NS
Triglycerides (mg dl^−1^)	93.5±6.7	115.7±8.6	<0.001
LDL-C (mg dl^−1^)	69.5±17.4	87.4±12.9	<0.001
ox-LDL (mU ml^−1^)	58.0±14.6	114.8±21.2	<0.001

Abbreviations: DPP-4, Dipeptidyl peptidase-4; EVOO, extra virgin olive oil; GIP, glucose-dependent insulinotropic polypeptide; GLP1, glucagon-like peptide-1; HDL, high-density lipoprotein; NS, not significant; Ox-LDL, oxidized LDL; low-density lipoprotein; LDL-C, LDL-cholesterol; HDL-C, HDL-cholesterol.
