# Supplementary material for: Extra virgin olive oil use is associated with improved post-prandial blood glucose and LDL cholesterol in healthy subjects
Source: Nutr Diabetes. 2015 Jul 20;5(7):e172–. doi: 10.1038/nutd.2015.23 (PMC4521177; doi:10.1038/nutd.2015.23)
Supplement: Supplementary Information [file nutd201523x1.doc]

**α-tocopherol concentration**

Oil samples (EVOO or corn oil) (100 μL) in amber-colour material to prevent the loss of vitamins, were supplemented with tocopheryl acetate as internal standard, deproteinized by the addition of ethanol, and extracted with hexane. Phase separation was achieved by centrifugation. The collected upper phase was evaporated and an aliquot analyzed using an Agilent 1260 Infinity Quaternary LC System equipped with an Eclipse Plus C18 column (4.6X100 mm)^1^.

**Total polyphenol content**

Total polyphenol content in EVOO and corn oil was determined by a modified Foline Ciocalteu colorimetric method^2^. Briefly, 0.1 mL of Foline Ciocalteu reagent was added to 0.02 mL of oils. After 5 min at room temperature, 0.05 mL of 20 g L-1 sodium carbonate was added and the reaction mixture was incubated at 37 oC for 20 min. The absorbance at 765 nm was measured using an Asys UVM 340 microplate reader (Biochrom, Holliston, USA) and compared with a gallic acid calibration curve (100-1000 mgL-1). Results were expressed as mg/ml of gallic acid equivalent (GAE). All experiments were performed in triplicate.

**Fatty Acids Methyl Ester determination**

50 mg of sample were extracted as described by Folch et al.^3^ and dried into a clear screw-capped glass tube. The percentages of fatty acids in samples were determined as methyl esters after vigorous methanolysis with 0.5N anhydrous HCl at 100 °C for 18h. After cooling to room temperature, the solution was extracted four times with hexane. The hexane phases containing the fatty acids methyl esters were collected, dried, and injected (0.5μl in Dichloromethane) into a SPB 2380 fused silica capillary column programmed at 5°C/min from 140 to 170°C for gas chromatographic analysis^4^.

**Oleuropein determination**

A 2.0 g amount of olive oil was accurately weighed into a 15mL tube. A 1 mL amount of the internal standard solution (syringic acid 0.015 μg/ml) was transferred to the samples. The sample tube was vortexed for 30 seconds and after 5 mL of methanol/water 80/20 (V/V) extraction solution were addede and again vortexed for exactly 1 minute before further extraction in the ultrasonic bath for 15 minutes at room temperature. Afterwards, the sample was centrifuged at 5,000 rev/min for 25 minutes. An aliquot of the supernatant phase was filtered through a 0.45 μm filter before injection into the HPLC system. 20 μL of samples were analysed using an Agilent 1260 Infinity Quaternary LC System equipped with ZORBAX Eclipse Plus C18 (4.6X250 mm, 5 μm). The separation is achieved using a ternary linear elution gradient with (A) water 0.2 % H3PO4 (v/v), (B) methanol and (C) acetonitrile.

**Acidity and Peroxide determination**

Acidity and the number of peroxide in oil samples were determinated by analitical titration.

**Table 1. Vitamin E and total polyphenols content in EVOO and Corn oil.**

| **Compounds** | **EVOO** | **Corn oil** | **P value** |
| --- | --- | --- | --- |
| Vitamin E (mg/Kg) | 75 | 18 | <0.001 |
| Total polyphenols (mg/L GAE) | 435 | 232 | <0.001 |
| Saturated Fatty Acid  (% m/m) | 18.67 | 13.0 | <0.001 |
| Mono-saturated Fatty Acid  (% m/m) | 66.6 | 29.0 | <0.001 |
| Poli-saturated Fatty Acid  (% m/m) | 13.6 | 58.0 | <0.001 |
| Peroxide (meq O_2_/kg) | <10 | <10 | <0.001 |
| Oleuropein (mg/Kg) | 4.0 | 0.01 | <0.001 |
| Acidity (%) | 0.34 | 1.1 | <0.001 |

**References**

1. Bieri JG, Tolliver TJ, Catignani GL. Simultaneous determination of alphatocopherol and retinol in plasma or red cells by high pressure liquid chromatography. *Am J Clin Nutr* 1979; **32**: 2143e9.

2. Mosca L, De Marco C, Visioli F, Cannella C. Enzymatic assay for the determination of olive oil polyphenol content: assay conditions and validation of the method. *J Agric Food Chem* 2000; **48**: 297e301.

3. Folch J, Lees M, Sloane-Stanley G.H. A simple method for the isolation and purification of total lipides from animal tissue. *J Biol Chem*. 1957; **226**: 497-509.

4. Cesare Alessandri, Pasquale Pignatelli, Lorenzo Loffredo, Luisa Lenti, Maria Del Ben, Roberto Carnevale et al. Alpha-Linolenic acid–rich wheat germ oil decreases oxidative stress and CD40 Ligand in patients with mild hypercholesterolemia. *Arterioscler Thromb Vasc Biol.* 2006; **26**: 2577-2578.
